# Hypoxia: Overview on Hypoxia-Mediated Mechanisms with a Focus on the Role of HIF Genes

**DOI:** 10.3390/ijms20246140

**Published:** 2019-12-05

**Authors:** Alexandru Andrei Tirpe, Diana Gulei, Stefana Maria Ciortea, Carmen Crivii, Ioana Berindan-Neagoe

**Affiliations:** 1 Faculty of Medicine, Iuliu Hatieganu University of Medicine and Pharmacy, 8 Victor Babes Street, 400012 Cluj-Napoca, Romania; altirpe@gmail.com (A.A.T.); sciortea@gmail.com (S.M.C.); 2 Research Center for Advanced Medicine-Medfuture, Iuliu Hatieganu University of Medicine and Pharmacy, 23 Marinescu Street, 400337 Cluj-Napoca, Romania; diana.c.gulei@gmail.com; 3 Department of Anatomy and Embryology, Iuliu Hatieganu University of Medicine and Pharmacy, 8 Victor Babes Street, 400012 Cluj-Napoca, Romania; 4 Research Center for Functional Genomics, Biomedicine and Translational Medicine, Iuliu Hatieganu University of Medicine and Pharmacy, 23 Marinescu Street, 400337 Cluj-Napoca, Romania; 5 Department of Functional Genomics and Experimental Pathology, The Oncology Institute “Prof. Dr. Ion Chiricuta”, 34-36 Republicii Street, 400015 Cluj-Napoca, Romania

**Keywords:** hypoxia, angiogenesis, metastasis, cancer metabolism, drug resistance

## Abstract

Hypoxia represents a frequent player in a number of malignancies, contributing to the development of the neoplastic disease. This review will discuss the means by which hypoxia powers the mechanisms behind cancer progression, with a majority of examples from lung cancer, the leading malignancy in terms of incidence and mortality rates (the frequent reference toward lung cancer is also for simplification purposes and follow up of the global mechanism in the context of a disease). The effects induced by low oxygen levels are orchestrated by hypoxia-inducible factors (HIFs) which regulate the expression of numerous genes involved in cancer progression. Hypoxia induces epithelial-to-mesenchymal transition (EMT) and metastasis through a complex machinery, by mediating various pathways such as TGF-β, PI3k/Akt, Wnt, and Jagged/Notch. Concomitantly, hypoxic environment has a vast implication in angiogenesis by stimulating vessel growth through the HIF-1α/VEGF axis. Low levels of oxygen can also promote the process through several other secondary factors, including ANGPT2, FGF, and HGF. Metabolic adaptations caused by hypoxia include the Warburg effect—a metabolic switch to glycolysis—and GLUT1 overexpression. The switch is achieved by directly increasing the expression of numerous glycolytic enzymes that are isoforms of those found in non-malignant cells.

## 1. Introduction

Hypoxia is a common feature of numerous malignancies. Despite the cancer dependency on hypoxia, the mechanisms of regulation are not identical. In the light of these aspects, the present review includes numerous examples from lung cancer (general classification of lung cancer: Small cell lung cancer (SCLC) which comprises 15% of the total lung cancers and non-small cell lung cancer (NSCLC) with a net percentage of 85% [1]. NSCLC is further divided into adenocarcinoma (AC)—40%, squamous-cell carcinoma (SCC)—30% and large-cell carcinoma (LCC)—10% of all lung cancers [2]) with the purpose of unifying the mechanisms in the context of a disease entity. Moreover, lung cancer remains the leading malignancy in terms of incidence and mortality according to GLOBOCAN 2018. This pathology was responsible for approximately 1.761 million deaths in 2018, with a staggering 2.094 million new cases in the same year [3,4]. Factors that contribute to these high numbers include late diagnosis and therapeutic failure caused by cancer progression and drug resistance. However, the paper also highlights regulatory axis that can frequently occur in other malignancies and not necessary in lung cancer. 

Intuitively, oxygen levels in tumors depend on several factors such as tumor size, stage, and the heterogeneity of the tumor [5]. HIF-1α, the principal regulator of hypoxia, mediates a large number of effects in tumor cells. First and foremost, hypoxia is able to induce epithelial-to-mesenchymal transition (EMT), process essential for cancer progression and metastasis [6]. Hypoxia activates several pathways that lead to EMT progression. These pathways will be addressed later in this review in the dedicated section. Furthermore, hypoxia is a known inducer of angiogenesis through HIF-1α modulation [7]. A dedicated section will explore the mechanism of hypoxia-induced angiogenesis. Additionally, under hypoxia, cancer cells present adaptative metabolic changes. Such changes, which will be addressed within the manuscript, can occur even in aerobic conditions and include the conversion of glucose to lactate and increased glucose uptake through the facilitative glucose transporters (GLUTs), phenomenon known as the Warburg effect [8].

## 2. Hypoxia-Inducible Factors (HIFs) are the Main Mediators of the Hypoxic Response

**Figure 1:**
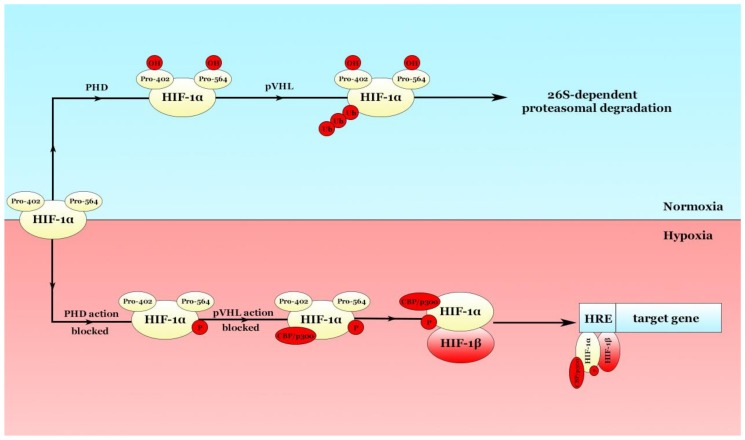
Hypoxia-inducible factor (HIF) regulation. HIF-1α molecule presents 2 proline (Pro) residues in the 402 and 564 positions. In normoxia, hydroxylation of the proline residues allows the pVHL to ubiquitinate the substrate, leading to a 26S-dependent proteasome degradation of the complex. Contrarily, in hypoxic environment, the action of PHDs is blocked, the substrate is phosphorylated, which then binds the CBP/p300 complex. After HIF-1β binds HIF-1α, the dimer attacks the HRE of the target gene, exhibiting specific effects.

Most of the hypoxic effects are mediated by a few types of heterodimers known as hypoxia-inducible factors (HIFs): HIF-1, HIF-2, and HIF-3 [9]. HIF-1 was discovered in 1992 by Semenza GL and his postdoc, Wang GL [10]. These heterodimers consist of an oxygen-sensible α-subunit and a stable β-subunit [11]. In normoxic conditions, the heterodimer is dissociated, prolyl hydroxylases (PHDs) hydroxylate two proline residues (Proline-402 and Proline-564) in the HIF-1α subunit [12] with consecutive ubiquitination of the HIF-1α by the von-Hippel-Lindau protein (pVHL) and proteasomal degradation of the subunit, canceling any effects HIF-1α may have. Kaelin WG’s team was the first to discover that VHL encodes a tumor suppressor protein, pVHL [13]. Mutations in the VHL gene can stabilize the HIFs in normoxic conditions by bypassing the ubiquitination checkpoint [14]. For example, VHL gene is rarely mutated in lung cancer. Lung ACs and SCCs have a small VHL mutation rate, with less than 1% in TGCA [9]. In contrast, in a low oxygen environment, PHDs are not able to hydroxylate the subunit and pVHL is unable to ubiquitinate its substrate, leading to the stabilization of HIF-1α [9]. Additionally, hypoxia is able to stabilize HIF-2α as well [15]. HIF-1α translocate into the nucleus and binds its heterodimer counterpart, HIF-1β, with consecutive generation of active HIF-1 which is ready to bind to hypoxia-responsive elements (HREs) and regulate the gene expression of various targets [16]. These mechanisms are depicted in Figure 1. The target genes control multiple functions such as angiogenesis (by both HIF-1 and HIF-2), erythropoiesis (solely by HIF-2) [17], cell growth, proliferation, metastasis via EMT, and also function as adaptive switches in metabolism. The degree of hypoxia-induced neovascularization is dependent on the expression level of two key players: VEGF-A and Angiopoietin-2 (ANGPT2). Some of the genes modulated by HIF-1α and HIF-2α are represented in Table 1. HIF-1α and HIF-2α have different expression profiles and different targets. For example, HIF-1α expression is ubiquitous, whilst HIF-2α expression is more localized in cells such as hepatocytes and endothelial cells [18]. 

**Figure 2:**
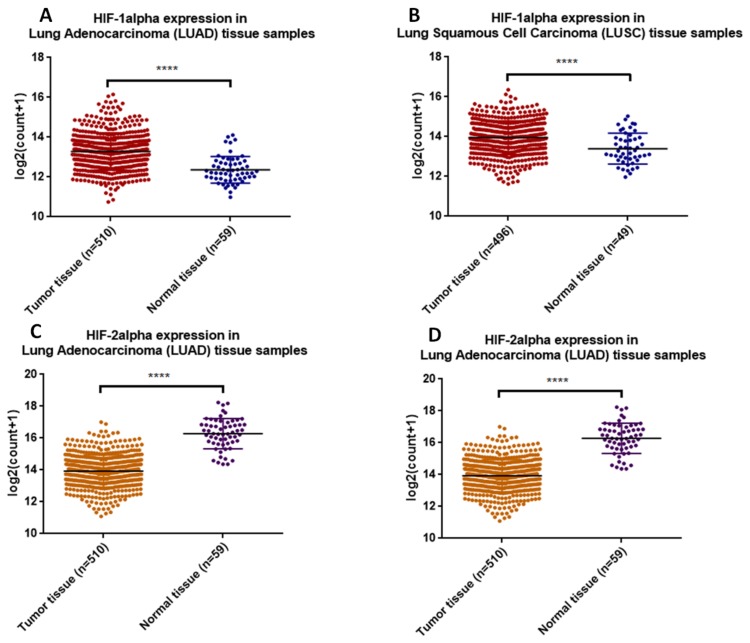
HIF-1α (**A,B**) and HIF-2α (**C,D**) expression in (**A,C***)* lung adenocarcinoma and (**B,D**) lung squamous cell carcinoma tissue samples from TCGA database (*data was download from dataset: Gene expression RNAseq–HTSeq—Counts for both LUSC and LUAD and represented as scatter plot, mean with SD).

HIFs are key players in a number of malignancies, including lung cancer. The prognostic role of HIF-1α and HIF-2α in NSCLC is a subject of controversy. While some authors reported that high HIF-1α expression may implicate poor prognosis [22] and shorter disease free survival, others found that HIF-2α overexpression rather than HIF-1α is a marker of poor prognosis in NSCLC [23]. TCGA analysis of HIF-1α and HIF-2α expression in lung adenocarcinoma (LUAD) and lung squamous cell carcinoma (LUSC) show an opposite expression of the two molecules. Specifically, while HIF-1α is upregulated in tumor tissue compared to normal adjacent one, the expression of HIF-2α is downregulated (Figure 2). These data suggest a possible predominant dependency of lung cancer on HIF-1α and not necessarily HIF-2α.

MicroRNAs (MiRNAs) are a class of small non-coding RNAs of 20–22 nucleotides involved in regulation of gene expression and modulation of different pathways [24]. MiRNAs are able to interact with the 3′-UTR of target genes and suppress their expression. Interactions with other regions such as the 5′-UTR and gene promoters have been described [25,26,27,28]. There are multiple miRNAs that modulate HIF-1α, including miR-18a, miR-155, miR-199a, miR-429, and miR-433 [29]. A more detailed description upon miRNAs involved in HIF-1α modulation in lung cancer can be consulted in Table 2.

## 3. Hypoxia and Metastasis: The Implication of the Hypoxic Effect on EMT Modulation

EMT is an essential process for cancer invasion with different mechanisms of regulation dependent on the type and subtype of malignancy. Generally, cells that undergo this transition lose expression of a number of epithelial markers such as epithelial cadherin (E-cadherin) and zonula occludens 1 (ZO-1) and increase the expression of mesenchymal markers such as neural cadherin (N-cadherin), fibronectin, and vimentin [6]. E-cadherin results from the translation of CDH1 and is a glycoprotein that enhances cell-to-cell adhesion and sustains cytoskeleton organization. Understandably, loss of E-cadherin contributes to cancer progression, invasion and metastasis [41,42]. Transcriptional repressors of E-cadherin in EMT include the zinc-finger E-box binding homeobox (ZEB), SNAIL and bHLH family factors such as TWIST, KLF8, and FOXC2. In a 2016 study on NSCLC specimens, Wei et al. found that TWIST expression was significantly associated with poor tumor differentiation, lymph node metastasis and TNM stage. A negative correlation was found between TWIST and E-cadherin and a positive correlation with vimentin and HIF-1α. Furthermore, in vitro experiments from the same team demonstrated increased levels of TWIST mRNA in NSCLC cells exposed to a hypoxic environment [43]. The opposite mechanism of EMT is mesenchymal to epithelial transition (MET), where cells go back to their non-invasive phenotype. However, these switches are actually taking place under a dynamic model, where in numerous cases the cells only achieve a hybrid state—epithelial/mesenchymal (E/M), having mixed characteristics between the epithelial and mesenchymal phenotype. Under the hybrid form, the cells can migrate together as clusters and can be found in the bloodstream of patients under the term of clusters of circulating tumor cells (CTCs) [44,45]. Early in 2011, it was shown for the first time the presence of hybrid CTCs that co-express keratin and vimentin in the blood of metastatic NSCLC patients [46]. Today, it is known that malignant CTCs are important contributors to prostate, lung, and breast metastasis and are associated with a poor clinical outcome [47]. These clusters are more resistant to apoptosis, contribute to drug resistance and also increase the metastatic potential in comparison with cells that are epithelial or mesenchymal [48]. Integrative analysis of mRNA, miRNAs, DNA methylation patterns, and proteomic data in lung adenocarcinoma showed that, besides the subsets of epithelial and mesenchymal cells, there is also an aggressive hybrid category that gathered molecular features from both phenotypes. Moreover, the aggressiveness of hybrid cells and the impact on survival was not attributed necessarily to the expression levels of E-cadherin or vimentin (the opposing mesenchymal marker), but rather to cytoskeletal and actin-binding proteins. Therefore, the study shows that the process of invasion can occur also in the presence of CDH1 expression [49]. 

A number of signaling pathways that drive EMT are modulated by HIFs. In general, EMT is regulated by an interplay between pathways such as transforming growth factor β (TGF-β), Wnt, Sonic Hedgehog (SHH) [50]. Essential pathways that block E-cadherin and are involved in EMT include the zinc-finger transcriptional factors SNAIL (SNAIL1), SLUG (SNAIL2), TWIST, the ZEB1 [51]. The promoter of the TWIST1 gene contains a HRE which can directly activate TWIST1 expression by binding either HIF-1 or HIF-2 [52,53].

TGF-β is an extensive inducer of EMT. Hypoxia stabilizes HIF-1α, which is known to mediate a TGF-β1-dependent pathway. The binding of TGF-β to its receptors TGFR1 and TGFR2 activates Smad2 and Smad3 through direct phosphorylation. These factors form a trimer with Smad4 and the complex is translocated into the nucleus, where a synergic action of the DNA binding transcription factors SNAIL, ZEB, and TWIST leads to the regulation of TGF-β target genes [41]. Smad complex is able to augment the activity of EMT TFs, with various targeted effects. For example, TGF-β can induce SNAIL1 expression via a Smad3-dependent transcription [54]. TGF-β activates the duo Smad3-Smad4 which is responsible for a number of events. Firstly, the duo interacts with SNAIL1 in order to suppress E-cadherin and occludin gene expression [55]. Secondly, by interacting with activating transcription factor 3 (ATF3) in order to suppress ID1 expression, Smad3-Smad4 increases TWIST expression [56]. PHD2 is downregulated by TGF-β1 via a SMAD-dependent pathway leading to increased levels HIF-1α [57] and creating a regulatory loop.

Notch is another EMT signaling pathway that can be induced by hypoxia. Sahlgren et al. showed that in the hypoxia/Notch/EMT axis, Notch can regulate SNAIL in two synergistic ways. First, N1ICD can be recruited to the SNAIL promoter, with a possible HIF-1α cooperation in the transcriptional complex and a direct upregulation of SNAIL. Another way in which Notch can regulate SNAIL is indirectly, by modulating LOX; HIF-1α can bind the LOX promoter, enhancing LOX protein production which increases SNAIL [58]. Several studies suggested that the expression of Notch pathway differs based on the histological type of lung cancer [59,60]. As such, Donnem et al. indicated that Notch1 expression was significantly lower in SCC compared to other NSCLC subtypes [59]. Contrarily, Li et al. found a significantly higher expression of Notch in lung SCCs compared to ACs. The same team found that 77% of NSCLC samples were positive for Notch1 compared to 47% in normal lung tissue. However, in SCLC, 68% of the samples were negative for Notch compared to 53% in normal lung tissue, rendering anti-Notch1 antibody therapy a negative feedback [60]. A meta-analysis realized by Yuan et al. found that Notch1 and Notch3 expression were positively correlated with NSCLC progression. The same meta-analysis indicated no statistical correlation between Notch1 and AC/SCC (pooled OR = 0.96, 95% CI: 0.75–1.22, *p* = 0.068 and I^2^ = 42.1%) and identified a greater possibility of lymph node metastasis (LNM) with higher tumor stages associated with Notch1 overexpression in NSCLC (pooled OR = 3.20, 95% CI: 1.81–5.65, *p* = 0.798 and I^2^ = 0.0%; pooled OR = 1.62, 95% CI: 1.00–2.62, *p* = 0.251 and I^2^ = 25.5%). Notch3 expression was linked with LNM but not with tumor size. Furthermore, Notch1 and Notch3 overexpression is a possible prognostic marker for overall survival (OS) [61]. 

Notch activity has also been identified in lung cancer stem cells (CSCs). Tumor cells with elevated Notch expression showed CSC properties—spheroid growth in cell cultures, a high level of chemoresistance, and implantation of a small number of cells into NOD/SCID mice can form tumors [62].

In SCLC, Notch1 signaling is usually absent, but has been reported after chemotherapy. Notch pathway is able to induce cell cycle arrest and apoptosis, a process largely involved in carcinogenesis [63,64,65].

### 3.1. The PI3k/Akt Pathway

Hong et al. determined that hypoxia, through HIF-2α is able to increase β-catenin expression via the PI3k/Akt pathway, suggesting that this signaling cascade is crucial in hypoxia-induced Wnt activation. Akt1 phosphorylation was also increased in hypoxic A549 cells; HIF-2α expressing lung cancer cells had a higher phospho-Akt1 expression compared to control or HIF-1α expressing cells [66].

In an in vitro experiment by Jin et al. on A549 and PC9 cells, the team demonstrated that netrin-1-mediated EMT in hypoxic conditions may be associated with the phosphoinositide 3 kinase/Akt pathway. The same effect of netrin-1 was not observable in the normoxic environment [67].

The Wnt/β-catenin signaling cascade is a well-known carcinogenic pathway, as cytoplasmic β-catenin can translocate into the nucleus and stimulate the transcription of several oncogenes by forming a complex with the T-cell transcription factor (TCF) [68]. The upregulation of PI3k/Akt pathway is essential for the hypoxia-mediated activation of the Wnt signaling cascade [66], as mentioned beforehand. According to Hong et al., hypoxia stabilizes β-catenin via a post-translational process and not through a de novo protein synthesis, followed by the activation of the Wnt cascade. In the case of lung cancer, the team determined that HIF-2α is the major factor that induces Wnt signaling, rather than HIF-1α. Increased β-catenin levels can induce morphological adaptations resembling those in EMT [66]. 

NME/NM23 nucleoside diphosphate kinase 1 (Nm23) is a key tumor suppressor involved in metastasis regulation and EMT; its deregulation has been associated with dysfunction in metastasis genes. Wu and team identified Wnt/β-catenin cascade as the chief mechanism in nm23-H1-mediated EMT in a hypoxic context in NSCLC [69]. 

Nuclear enriched abundant transcript 1 (NEAT1) is a long non-coding RNA (lncRNA) located on chromosome 11 whose abnormal expression has been identified in a number of cancers and is involved in the miR-101-3p/SOX9/Wnt/β-catenin axis. Choundhry et al. reported that high HIF-2α levels are a contributing factor to the activation of NEAT1 [70], which functions as an oncogene in NSCLC, according to Kong et al. [71]. The latter team’s in vitro results showed that HIF-2α and NEAT1 levels were higher in hypoxic environment. Furthermore, overexpression of HIF-2α upregulated NEAT1 expression stimulated EMT in hypoxic conditions. In the same study, Kong and the team identified miR-101-3p as a potential target of NEAT1—NEAT1 downregulation increased miR-101-3p expression, rendering it as a direct target of NEAT1. Moreover, miR-101-3p targets SOX9 which bridges the pathway to Wnt/β-catenin. Several studies in different types of cancer showed the connection between SOX9 and the Wnt/β-catenin [72,73,74]. As a result, the oncogenic lncRNAs NEAT1 promotes NSCLC progression by activating the miR-101-3p/SOX9/Wnt/β-catenin axis.

### 3.2. Nuclear Factor-κB (NF-κB)

NF-κB is a ubiquitous transcription factor involved in multiple physiological and pathological processes, including cancer. In 1994, Koong et al. were the first to report that hypoxia is able to activate NF-κB [75]. An extensive bidirectional crosstalk between HIF and NF-κB has also been described [76], highlighting the influence that hypoxia may have on the subjacent pathways. NF-κB subunits are located in cytoplasm in an inactive form, allowing a rapid response following an activating stimulus [76]. Furthermore, induction of NF-κB by hypoxia can be achieved via an IkappaB kinase (IKK)- and TAK1-dependent manner, but independent of *oxygen sensors* PHD1, 2, 3, and HIF-1α. The main mechanism of this HIF-independent activation consists of activation of TAK1 and IKK by the Ca^2+^-dependent calcium/calmodulin-dependent kinase (CaMK)2. This activation of CaMK2 precedes the inhibition of PHD and HIF-1α *sensors* [77]. In hypoxic environment, Ca^2+^ is released mainly by two pathways: First, hypoxia increases reactive oxygen species (ROS) production in the mitochondria with consecutive release of Ca^2+^ from the endoplasmic reticulum (ER) via the ryanodine receptors (RyRs). Second, calcium can be released from the ER via the oxidase pathway. A crosstalk has been identified between these two pathways [78]. Of note is the connection of CaMK with NF-κB as a result of Toll-like receptor (TLR) signaling, as CaMK2 was found to directly bind and activate TAK1 [79]. IKK activation in hypoxic conditions requires TAK1. Culver and team have also identified that NF-κB transcription activity can be modulated by hypoxia [77]. The main molecules in the NF-κB classical signaling pathway include the phosphorylated IKK and phosphorylated p65. The latter translocates into the nucleus and is in charge of activating the transcription of downstream genes by binding to specific sequences in their promoter [80]. In a recent in vitro study by Wang et al., BAY11-7082, a NF-κB pathway suppressor, inhibited EMT and cancer cell stemness in hypoxic conditions in the SPC-A1 and H1299 NSCLC cell lines. The EMT-related markers SNAIL, N-cadherin, and vimentin were found to be downregulated, whilst E-cadherin was upregulated, indicating loss of EMT function. Thus, Wang and team proposed that NF-κB signaling cascade may be involved in EMT in NSCLC [81].

**Figure 3:**
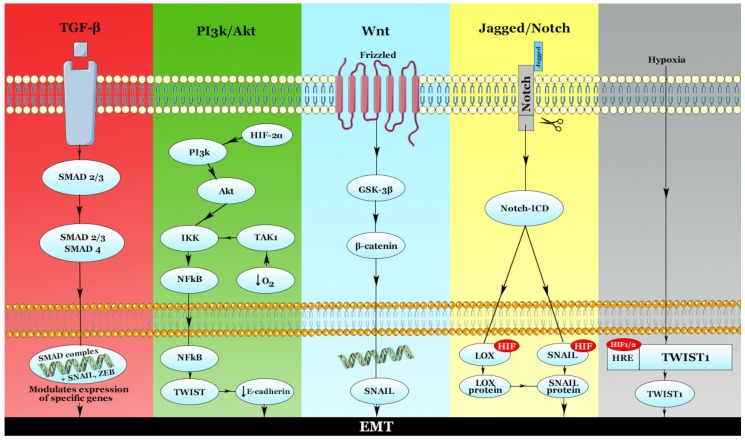
Pathways involved in hypoxia-dependent epithelial-to-mesenchymal transition (EMT). The main pathways that mediate EMT activation are TGF-β, PI3k/Akt, Wnt, and Jagged/Notch. The TGF-β pathway is SMAD-mediated; the SMAD complex binds specific DNA regions along with transcription factors such as SNAIL and zinc-finger E-box binding homeobox (ZEB) in order to modulate EMT-related gene expression. Another crucial pathway in hypoxia-mediated EMT is PI3k/Akt. HIF-2α is able to induce this pathway, with a concomitant activation of NF-κB/TWIST, and a downregulation of E-cadherin. Obviously, this downregulation leads to loss of cell-to-cell junctions and promotes EMT. The Wnt pathway, as well as the Jagged/Notch pathway, induces EMT in a SNAIL-dependent manner. Hypoxia can also stimulate EMT directly—HIF-1/2 can bind the HRE of the TWIST1 gene in order to promote its expression, thus leading to EMT.

These hypoxia-inducible pathways that modulate EMT are summarized in Figure 3.

## 4. Hypoxia and the Genesis of New Blood Vessels: The Modulation of Angiogenesis by Hypoxia

Angiogenesis is an essential treat for cancer progression. Tumor growth beyond 2 to 3 mm^3^ requires the genesis of new blood vessels in order to sustain the progressive increase in size [49,82]. Hypoxia is able to directly drive angiogenesis in lung cancer by activating the HIF-1α/VEGF signaling pathway [83]. HIF-1, the hypoxic master regulator, binds to the HRE in the promoter region of the key driving entity in angiogenesis—VEGF [84]. The signaling cascade responsible for the VEGF regulation is the PI3K/Akt pathway [85]. Other angiogenic growth factors stimulated by hypoxia include ANGPT2 through HIF-2α [7], placental growth factor (PlGF), platelet-derived growth factor beta (PGDF-β), stromal cell-derived factor-1α (SDF-1α), and stem cell factor (SCF). In order for these growth factors to induce their effects, they need to bind to the corresponding receptors on the surface of endothelial cells or smooth muscle cells. A summary of these factors and their corresponding receptors can be viewed in Table 3. Direct targets of HIF-1α include VEGF, ANGPT2, SDF1, and SCF genes [84,86,87,88]. ANGPT2, through its antagonizing action on TIE2, destabilizes existing vessels, thus partly regulating vascular remodeling [7]. ANGPT1 is a relative of ANGPT2 expressed in endothelial cells and pericytes [89]. ANGPT1 is induced by hypoxia and has agonist activity on the TIE2 receptor, promoting angiogenesis by mediating the interactions between endothelial cells and pericytes in maturing vessels [7,90]. Hypoxia also increases the well-known pro-angiogenic factors fibroblast growth factor (FGF) which promotes EC proliferation and induces migration, and the hepatocyte growth factor (HGF) with an essential role in vascular permeability [83]. A number of studies demonstrated that HIF-1α and HIF-2α have a complementary action in angiogenesis: HIF-1α drives vessel growth whilst HIF-2α enhances vessel maturation [89,91].

Hypoxia is also able to target several miRNAs involved in angiogenesis. One such miRNA is endothelial miR-210 whose HIF-related activation enhances endothelial tube formation and migration of the ECs through Ephrin-A3 downregulation [93]. Macrophages targeted by hypoxic cancer cell-derived extracellular vesicles (EV) containing miR-103a will induce the production of high levels of pro-angiogenic factors such as VEGF and ANGPT-1, promoting angiogenesis. MiR-103a EVs decrease PTEN expression in monocytes in the tumor microenvironment (TME). Consecutively, there is an excessive activation of the PI3k/Akt pathway which is involved in angiogenesis [94]. Hypoxia-responsive miRNAs (HRMs) can be induced by both HIF-1α and HIF-2α under hypoxia. HRMs such as Let-7 and miR-103 can target argonaute 1 (AGO1) and release VEGF mRNA which is secluded in the miRISC. Hypoxia decreases AGO1-associated miR-15 and miR-29 which have been demonstrated to target VEGF as the silencing effect requires miRISC and a functional AGO1, plus the miRNA-mRNA duplex [95].

Another important route of metastasis is represented by the lymphatic system, where the mechanistically synonym process is called lymphangiogenesis. Hypoxia-induced HGF can promote lymphangiogenesis either directly through activating its receptor, c-Met, or indirectly via the VEGF-C/VEGFR-3 signaling pathway [96]. The VEGF-C and VEGF-D have been established as central players in the development of lymphangiogenesis through the interaction with VEGFR3 in lymphatic endothelial cells [97,98,99,100]. This receptor is a transmembrane tyrosine kinase receptor type and is normally expressed only during embryonic development in lymphovascular progenitors [101]. However, in cancer, the expression of VEGFR3 is forced in adult endothelial cells and lymphatic endothelial cells in order to contribute to the development of angiogenesis and lymphangiogenesis [102,103]. Meantime, the same molecules can also bind VEGFR2 with eventual direct effects upon the proliferation and migration of endothelial cells [104,105]. However, the specificity of the lymphangiogenic cascade is achieved through the inability of the VEGF-C and VEGF-D to actually activate VEGFR1 and VEGFR2 in endothelial cells. The two molecules have also been significantly associated with HIF-1α expression in various cancers through immunohistochemical techniques [106,107]. Molecular analysis showed that hypoxic conditions determines the transcription at mRNA level of VEGF-C and VEGF-D in lymphatic endothelial cells [108], where in macrophages, HIF-1α can manage the expression of VEGF-C via a hypoxia response element (HRE) sequence located within the promoter of the VEGF-C gene [109]. The expression of the same gene in hypoxia can be induced also independently of HIF-1α regulation through a mechanisms dependent of an internal ribosome entry site [110]. The global regulation in low oxygen concentration in cancer is also developed at the level of tumor associated fibroblasts and immune cells that can also express preferentially VEGF-C and VEGF-D with the final purpose of contributing to the malignant progression [111,112]. The strong implications of VEGF-C and VEGF-D in cancer development has been noted in studies correlating their expression level with metastatic spreading [113] and also in preclinical testing involving inhibition of their expression that concluded with limitation of lymph node and distant dissemination [114]. The involvement of VEGF-A in lymphangiogenesis was highlighted through experimental inhibition of VEGFR3 that still not completely abrogated the pathological development of the lymphatic system, showing that VEGF-A is contributing through the VEGF-A-VEGFR2 axis [115,116]. Moreover, it has been shown that lymphatic endothelial cells express also VEGFR2 and not only VEGFR3 [117]. Even so, the direct link between HIFα and VEGF-A, that is strongly explored in angiogenesis, is lacking in lymphangiogenesis [101]. 

## 5. Metabolic Adaptations Induced by Hypoxia in Cancer

Cells in normoxic environment convert glucose into pyruvate which enters the Krebs cycle and undergoes oxidative phosphorylation in the mitochondria with a final production of adenosine triphosphate (ATP). However, tumor cells display an increase in glucose consumption and an important metabolic switch to glycolysis; hence, the pyruvate is converted into lactate. This switch to glycolysis in tumor cells is present even in aerobic conditions and is called the Warburg effect [8]. The glycolytic process in tumors may be driven by active HIF-1α, independently of hypoxia [118], as multiple enzymes in charge of the metabolic switch are induced by HIF-1α [119]. KRAS, a well-known mutated oncogene in lung cancer is an inducer of aerobic glycolysis [120].

HIF-1α increases the expression of a large number of glycolytic enzymes which are isoforms to those found in non-malignant cells. In lung cancer, HIF-1α modulates the expression of several isoforms, including GLUT1 and GLUT3, hexokinase (HK) II, hexosephosphate isomerase (HPI), phosphofructokinase (PFK)-L, aldolase (ALD)-A, triosephosphate isomerase (TPI), glyceraldehyde-3-phosphate dehydrogenase (GAPDH), phosphoglycerate mutase (PGAM)-B, enolase (ENO)-α, pyruvate kinase (PYK)-M2 [121]. HIF-1α is a known inducer of pyruvate dehydrogenase kinase (PDK) which deactivates pyruvate dehydrogenase (PDH) by a phosphorylation mechanism, thus inhibiting the entrance of pyruvate in the tricarboxylic acid (TCA) cycle and its transformation into acetyl-CoA [122]. The same hypoxic master regulator induces lactate dehydrogenase-A (LDHA) enzyme which converts pyruvate into lactate [118]. 

In order to survive and adapt to the conditions derived from the metabolic alterations, tumor cells need to export the excess acidic ions outside their environment. Hypoxia, through HIF-1α has been shown to modulate the expression of the monocarboxylate transporter (MCT) 4, a H^+^/lactate co-transporter [123]. Furthermore, the sodium-hydrogen antiporter (NHE) 1 is modulated by HIF-1α and has the capability to excrete the intracytoplasmatic H^+^ [124]. HIF-1α induces carbonic anhydrase IX (CAIX) and carbonic anhydrase XII (CAXII) which convert CO_2_ into carbonic acid which dissociates into HCO_3_^–^ and H^+^. The basic HCO_3_^−^ is then transported inside the cell, increasing the intracellular pH. Thus, the external tumor microenvironment is acidotic and has been correlated with a poor prognosis [119].

As hypoxic tumors’ energetic demands lie on glycolysis, a process with smaller yield compared to oxidative phosphorylation, the cellular glucose intake must increase in order to satisfy these demands. Glucose is vehiculated into the cell via the GLUT family. Kurata et al. identified GLUT1 as overexpressed in primary lung cancer compared to other transporters from the GLUT family [125]. GLUT1 overexpression might be connected with the distance from stromal blood supply, indicating an association with tumor hypoxia [118]. Hypoxia induces high levels of GLUT1 by suppression of oxidative phosphorylation and upregulation of the hypoxic master regulator, HIF-1α [126]. In NSCLC, there is a positive correlation between GLUT1 expression and the EGFR and KRAS oncogenes; GLUT1 overexpression is also correlated with an aggressive phenotype [127]. In an in vivo study by Micucci et al., the hyperglycemic state induced by streptozotocin accelerated lung cancer progression [128]. Hyperglycemia induces reactive oxygen species (ROS) production by increasing oxidative stress, decreases mitochondrial function and tilts the metabolic processes to glycolysis [120,129].

**Figure 4:**
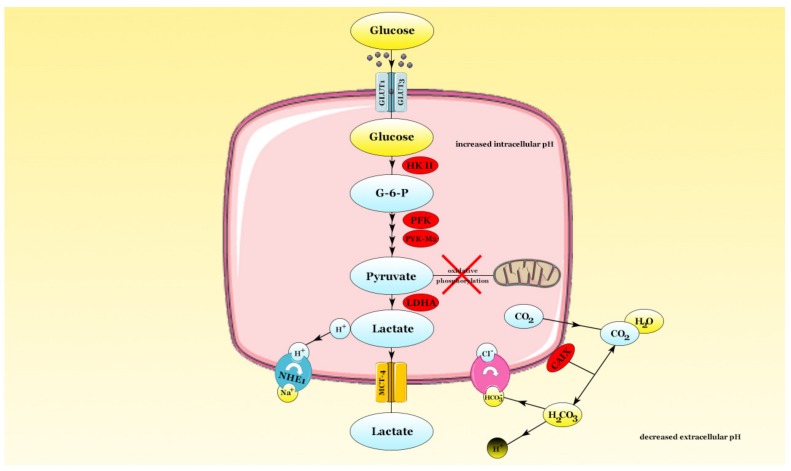
The metabolic switch to glycolysis. Glucose enters the cell via the GLUT1 and GLUT3 transporters. In the intracellular environment, glucose is converted into glucose-6-phosphate and then into pyruvate following a succession of metabolic reactions catalyzed by HIF-induced enzymes HK II, PFK, PYK-M2. Consequently, pyruvate does not enter oxidative phosphorylation and is converted into lactate through the action of lactate dehydrogenase-A (LDHA). Lactate is transported towards the extracellular compartment through the MCT-4 transporter, whilst CO_2_ resulted from the metabolic conversions diffuses extracellular. The membrane-bound carbonic anhydrase IX (CAIX) synthesizes carbonic acid which dissociates in bicarbonate and H+. The HCO3- is transported into the cell, increasing the intracellular pH, whilst the H+ decreases extracellular pH. The extracellular pH is further decreased by lactate-derived H+ which antiports Na+ through the NHE1 antiporter. The enzymes colored in red are HIF-1α-induced.

The main metabolic adaptations along with the hypoxia-induced enzymes are depicted in Figure 4.

## 6. Conclusions

Cancer development is driven by a large number of factors that are interconnected within different mechanisms. One such factor is hypoxia. In this review, we have shown that hypoxia plays an important role in cancer progression by sustaining vital mechanisms such as EMT, angiogenesis, and metabolic adaptations. 

First and foremost, we have shown that hypoxia is able to drive EMT, a major factor in cancer metastasis. HIFs can activate EMT through various pathways, including TGF-β, Notch, PI3k/Akt, Wnt/β-catenin, and NF-κB. The loss of E-cadherin expression contributes to invasion and metastasis [41,42] and is achieved through hypoxia-mediated transcriptional repression. Another process that is regulated by hypoxia and leads to cancer progression is angiogenesis. As we have reported, the HIF-1α/VEGF axis is the primary mechanism through which hypoxia activates angiogenesis [83]. Tumor growth beyond 2 to 3 mm^3^ requires additional blood supply [82]. Thus, hypoxia-mediated angiogenesis sustains cancer growth. Another mechanism modulated by hypoxia was reported to be the metabolic switch from oxidative phosphorylation to glycolysis [130]. Moreover, therapeutic resistance can be induced in a hypoxic manner. This feature is currently one of the most investigated areas within health-related research due to numerous cases of non-responsive or recurrent patients. The mechanisms behind this feature are vast, but mainly consist in increased DNA repair ability, apoptosis inhibition and variations in cell metabolism, as well as HIF-1α-mediated autophagy [131,132,133,134]. The implication of these resistance mechanisms are vast and need to be approached in a different article.

Targeting the HIF pathway in cancer is quite challenging due to the heterogeneity of the target genes and also their different regulation dependent on the cancer types [135]. Currently, there are several therapeutics in preclinical testing able to block HIF activity with the intention of limiting the tumor development, angiogenesis, and metastasis. Preliminary screening research is on the way for identification of small molecules able to interact with the activity of HIF-1; NCI DIVERSITY SET of small molecules indicated that TOPOISOMERASE I INHIBITORS are also reducing the HIF-1α expression through a yet-unknown mechanism [136]. Other small molecules like YC-1, thioredoxin inhibitors, 17-AAG, and 2ME2 were associated with the capacity of reducing the HIF-1α levels together with limitation of tumor growth and angiogenesis in vivo. However, none of these small molecules were designed for specific targeting of HIF-1α, fact that hampers the translational switch toward the patients due to difficulties in controlling the level of action and selection of the targeted cohort [137]. There are also clinical approved drugs that were shown to interfere with HIF-1α activity (non-specific HIF-1α inhibitors): Angiotensin-2 receptor blocker, digoxin or metformin [135,138,139]. Prostate cancer patients prescribed with one of these three therapies have been associated with decreased risk of cancer progression [140]. Considering the past and ongoing preclinical studies that have constantly shown the impact of HIF genes upon cancer progression, the development of specific HIF inhibitors seems like a realistic option for novel oncology therapeutics. Moreover, these inhibitors could function in synergy with drugs developed for inhibition of angiogenesis: The strategy behind is represented by the idea that cancer cells adapted to hypoxic condition could survive also after anti-angiogenesis therapy. Moreover, it has been shown that hypoxic cells are more resistant to chemotherapeutics and radiation and have increased invasive abilities correlated with reduced patients’ survival [137]. 

Concluding, hypoxia acts as a ‘bad cop’, rather than a ‘good cop’ in several malignancies. It mediates a large number of mechanisms that promote cancer progression/therapeutic resistance and ultimately an increased mortality. 

## Figures and Tables

**Table 1:** Various HIF-1α and HIF-2α targets organized by the modulated process. Gene targets were compiled from [19,20].

HIF-1α-Modulated Entities			HIF-2α-Modulated Entities		
Angiogenesis	Cell Survival and Proliferation	Metabolism	Angiogenesis, Blood Vessel Remodeling	Erythropoiesis	Cell Cycle Progression
VEGF-A ^1^	Insulin-like growth factor 2	GLUT1 ^1^	ANGPT2	EPO ^2^	CCND1
TGF-β3	Insulin-like growth factor binding protein (IGF-BP)-1	GLUT3			
	IGF-BP3	Hexokinase (HK) 1			
	c-Myc	HK 2			

^1^ According to Keith et al., 2012, VEGF-A and GLUT1 can be modulated by both HIF-1α and HIF-2α [19]. ^2^ The original article by Ratcliffe’s laboratory found that EPO mRNA levels were oxygen-dependent [21].

**Table 2:** MiRNAs targeting HIF-involved molecules in lung cancer.

MicroRNA	miRNA Expression in Cancer	Target	Effect	Ref
**miR-23a**	Upregulated	PHD1, PHD2 ZO-1	MiR-23a inhibits PHD1, PHD2 leading to HIF-1α stabilization in endothelial cells. MiR-23a also inhibits ZO-1, increasing vascular permeability and migration.	[30]
**miR-370**	Downregulated	HIF-1α EGFR	In a study by Liu et al., miR-370 overexpression decreased EGFR and HIF-1α expression and reduced the extracellular single-regulated kinase (ERK)1/2 and AKT phosphorylation. As such, miR-370 could inhibit NSCLC growth, angiogenesis and metastasis.	[31]
**miR-622**	Downregulated	HIF-1α	In a study by Cheng et al., miR-622 targeted the 3′-UTR of HIF-1α mRNA and downregulated its expression, with a consecutive decrease in mesenchymal protein levels and an inhibition in cell migration and invasion in vitro.	[32]
**miR-18a-5p**	Decreased after radiation exposure; Increased in the plasma of patients from the radiosensitive group compared to radioresistant one	HIF-1α Ataxia teleangiectasia mutated (ATM)	Results by Chen et al. show that miR-18a-5p downregulates HIF-1α and ATM expressions and increases sensitivity to radiotherapy in lung cancer cells and in CD133+ stem-like cells.	[33]
**miR-18**	*Screening for miRNAs targeting HIF-1α*	HIF-1α	MiR-18 downregulates HIF-1α mRNA and protein levels.	[34]
**miR-549**	*Screening for miRNAs targeting HIF-1α*	HIF-1α	MiR-549 downregulates HIF-1α transcriptional activity, mRNA and protein levels.	[34]
**miR-200c**	*Screening for miRNAs targeting HIF-1α*	HIF-1α	MiR-200c downregulates HIF-1α transcriptional activity, mRNA and protein levels.	[34]
**miR-214**	Upregulated	ING4	In a study by Li et al., miR-214 upregulated HIF-1α and VEGF levels by targeting ING4 in an in vitro experiment on lung cancer cells.	[35]
**miR-31-5p**	Upregulated	Factor inhibiting HIF-1α inhibitor (FIH)	Overexpressed miR-31-5p directly targets FIH, resulting in high HIF-1α levels, upregulated aerobic glycolytic genes, thus enhancing the Warburg effect.	[36]
**miR-155-5p**	Radiation controlled expression—increased after radiotherapy	HIF-1α	Radiation therapy was found to upregulate miR-155-5p, which consecutively inhibited HIF-1α and suppressed the NSCLC cells malignancy.	[37]
**miR-519c**	Increased in patients with better prognosis	HIF-1α	A study by Cha et al. found that an overexpression of miR-519c in mice induced low HIF-1α levels, suppressed angiogenesis, growth and metastasis. Consequently, overexpression of miR-519c in cancer patients produced a better prognosis.	[38]
**miR-182**	Context dependent expression—increased in 95C NSCLC cell line and decreased in A549 NSCLC cell line	FIH1	A study by Wang et al. on NSCLC cells identified FIH as a target of miR-182. FIH silencing leads to an overexpression of HIF-1α with a consecutive metabolic switch to glycolysis.	[39]
**miR-17-92 cluster**	Amplification and overexpression	HIF-1α	A study by Taguchi et al. identified HIF-1α as a target of the miR-17-92 cluster under normoxic conditions, suggesting that this cluster may play a role in regulation of basal levels of HIF-1α in normoxia. The same authors suggested the possible existence of a c-myc, HIF-1α and miR-17-92 circuit that would be involved in cancer cell proliferation under normoxia.	[40]

**Table 3:** Pro-angiogenic growth factors/molecules stimulated by hypoxia and their corresponding receptors. (Table after Rey et al., 2010, Cardiovasc Res [92]).

Growth Factor/Molecule	Receptor
VEGF	VEGFR1, VEGFR2
ANGPT1 ^1^	TIE2
ANGPT2 ^2^	TIE2
PlGF	VEGFR1
PDGF-β	PDGFR-α, PDGFR-β
SDF1	CXCR4
SCF	C-KIT

^1^ ANGPT1 has agonist activity on TIE2. ^2^ ANGPT2 has antagonist activity on TIE2.
